# Fabrication of a Spiral Microfluidic Chip for the Mass Production of Lipid Nanoparticles Using Laser Engraving

**DOI:** 10.3390/mi16050501

**Published:** 2025-04-25

**Authors:** Inseong Choi, Mincheol Cho, Minseo Song, Byeong Wook Ryu, Bo Mi Kang, Joonyeong Kim, Tae-Kyung Ryu, Sung-Wook Choi

**Affiliations:** 1 Biomedical and Chemical Engineering, Department of Biotechnology, The Catholic University of Korea, 43 Jibong-ro, Wonmi-gu, Bucheon-si 14662, Gyeonggi-do, Republic of Korea; 2 Department of Neurology, Institute for Cell Engineering, Johns Hopkins University School of Medicine, Baltimore, MD 21205, USA

**Keywords:** perfluoropolyether, microfluidics, lipid nanoparticles, mass production, laser engraving

## Abstract

A spiral microfluidic chip (SMC) and multi-spiral microfluidic chip (MSMC) for lipid nanoparticle (LNP) production were fabricated using a CO_2_ laser engraving method, using perfluoropolyether (PFPE) and poly(ethylene glycol) diacrylate as photopolymerizable base materials. The SMC includes a spiral microchannel that enables rapid fluid mixing, thereby facilitating the production of small and uniform LNPs with a size of 72.82 ± 24.14 nm and a PDI of 0.111 ± 0.011. The MSMC integrates multiple parallel SMC structures, which enables high-throughput LNP production without compromising quality and achieves a maximum production capacity of 960 mL per hour. The LNP fabrication technology using SMC and MSMC has potential applications in the pharmaceutical field due to the ease of chip fabrication, the simplicity and cost-effectiveness of the process, and the ability to produce high-quality LNPs.

## 1. Introduction

The COVID-19 pandemic has highlighted the significance of mRNA vaccines in the biomedical and pharmaceutical fields, leading to the development of lipid nanoparticles (LNPs) as an efficient delivery vehicle for mRNA. LNPs have excellent biocompatibility, protect mRNA from degradation, and enhance intracellular delivery efficiency [1,2,3]. The major cargo of LNPs, mRNA, has a well-defined chemical structure and offers manufacturing advantages over traditional vaccines, such as inactivated vaccines, live vaccines, and recombinant protein vaccines. This makes it particularly suitable for responding to rapidly mutating viral infectious diseases. Moreover, LNP-based formulations are being actively investigated not only for use in vaccines but also for cancer therapy and other disease treatments [4,5,6,7].

LNPs larger than 200 nm are primarily cleared by the spleen, liver, and reticuloendothelial system, hindering their cellular uptake via endocytosis. Therefore, particle size is a critical factor in determining the therapeutic activity of LNPs [8,9,10]. Additionally, larger LNPs have a relatively smaller surface area, which results in faster drug release. Thus, achieving a uniform particle size is crucial for maintaining the stability and controlled release of LNPs.

One of the most-used methods of LNP production is ethanol injection, wherein lipid components dissolved in water-miscible solvents such as ethanol or isopropyl alcohol are introduced into an aqueous solution containing nucleic acids. The ethanol injection method is based on nanoprecipitation, which is induced by the solubility changes driven by intermolecular diffusion between ethanol and the aqueous phase [11]. Since homogeneous mixing plays a critical factor in LNP fabrication, microfluidic chips have emerged as a major platform for LNP production due to their advantages, including short mixing times, a high mixing efficiency, and excellent reproducibility. Microfluidic chips designed for LNP production incorporate specialized microstructures such as staggered herringbone, serpentine, zig-zag, and post-obstacle patterns to facilitate fluid mixing without requiring additional external equipment [12,13,14,15]. These passive microfluidic mixers reduce the physical shear forces applied to the fluid, thereby preventing drug degradation within the LNPs. They also offer superior reproducibility and miniaturization [16,17]. However, the inherently low throughput of microfluidic chips due to their small internal volume remains a limitation, prompting ongoing research efforts to enhance production scalability. Sarah J. Shepherd et al. developed a parallelized microfluidic device (PMD) using PDMS material for the large-scale production of LNPs. The PMD successfully produced LNPs at a rate exceeding 10 L/h without a reduction in quality, utilizing 128 staggered herringbone micromixers [18]. Masatoshi Maeki et al. [19] fabricated a microfluidic device with a repetitive baffle structure using PDMS and glass, arranging eight of these devices to produce LNPs at a rate of 160 mL/min. However, the soft lithography technique used for PDMS processing requires expensive equipment and is complex, while the wet etching method employed for glass fabrication also presents challenges due to its complexity and limited accessibility [19]. Also, photolithography needs an extremely clean environment and complex system for UV exposure and to clean the photoresists.

The laser engraving method uses a CO_2_ laser and is based on the use of the photothermal effect to create micro-patterns. Laser engraving equipment is cost-effective and accessible, requiring minimal expertise for use [20,21,22,23,24]. However, the method has certain limitations, such as a limited range of usable materials, rough surfaces in the fabricated microchannels, a low resolution (~100 μm), and difficult-to-design 3D structures [24,25,26,27,28].

In previous studies, we reported a simple method for fabricating microfluidic devices using CO_2_ laser engraving. We successfully fabricated microfluidic devices with precise fluid control and hydrophilic surfaces using Perfluoropolyether (PFPE) and Polyethylene glycol diacrylate (PEGDA) as base materials. Unlike PMMA, which is commonly used for laser engraving, the PFPE elastomer exhibits low surface energy, non-toxicity, and excellent solvent resistance, making it an ideal material for microfluidic device fabrication [29].

In this study, to expand the range of applications, a Spiral Microfluidic Chip (SMC) and a Multi-Spiral Microfluidic Chip (MSMC) were fabricated using laser engraving. SMC and MSMC contain specialized spiral microchannels that facilitate rapid fluid-mixing, enabling the precise control of LNP size. Additionally, the MSMC, which integrates multiple parallelized spiral microchannels, allows for the continuous production of small and uniformly sized LNPs at a maximum flow rate of 960 mL/h. The MSMC fabrication technology using laser engraving offers excellent accessibility and scalability, making it a promising platform for the manufacture of LNPs.

## 2. Materials and Methods

### 2.1. Materials

Polydimethylsiloxane (PDMS) elastomer and curing agent (Sylgard^®^ 184) were purchased from Sewang Hitech (Gimpo, Republic of Korea). PFPE (Fluorolink^®^ MD 700, Solvay, Bruxelles, Belgium), polyethylene glycol diacrylate (PEGDA, M_n_ = 700, Sigma-Aldrich, St. Louis, MO, USA), and 2-hydroxy-2-methylpropiophenone (Darocur^®^ 1173, Sigma-Aldrich, St. Louis, MO, USA) were used for the fabrication of SMC and PSMC. 1,2-dipalmitoyl-sn-glycero-3-phosphocholine (16:0 PC, DPPC) and cholesterol were purchased from Avanti Polar Lipids (Alabaster, AL, USA) to produce LNPs. Phosphate-buffered saline (PBS) was purchased from Welgene (Gyeongsan, Republic of Korea).

### 2.2. Fabrication of Microfluidic Chip

The spiral microfluidic chip (SMC) consists of two layers of polymer sheets composed of PFPE and PEGDA and one PDMS layer for tube-fitting. The first layer is a PFPE-PEGDA polymer sheet with a spiral-shaped microfluidic channel engraved via laser engraving, and the second layer is a PFPE-PEGDA polymer sheet with two inlets and one outlet hole. To fabricate the PFPE-PEGDA polymer sheet, the PFPE solution and the PEGDA solution were mixed at a weight ratio of 4:1, and then 0.1% Darocur^®^ 1173 was added. After degassing in a vacuum chamber, the prepared PFPE-PEGDA solution was poured into the mold with 0.5 mm thickness using two glass plates. The mold was placed in a UV curing system (Inno cure 850, Lichtzen, Gunpo, Republic of Korea) and cured for 100 s to obtain the PFPE-PEGDA polymer sheet. The fabricated polymer sheet was cut to a size of 15 × 40 mm to form the first layer, and a microfluidic channel was engraved using a CO_2_ laser (ML-4040, Machine Shop, Pyeongtaek-si, Republic of Korea) to fabricate the first layer. The microfluidic pattern was designed using Autodesk Fusion v.2.0.21508 (Autodesk, CA, USA) and Adobe Illustrator software CS5 (Adobe Systems, CA, USA). The wide inlet channel was engraved at a laser power of 9.3% and a nozzle speed of 40.0 mm/s, while the thin spiral channel was engraved with a laser power of 6.7% and a nozzle speed of 3.5 mm/s. The two inlets and an outlet in the second layer were punched using a CO_2_ laser with a laser power of 11.3% and a nozzle speed of 5.0 mm/s. The engraved PFPE-PEGDA sheet was washed with ethanol. The top PDMS layer was fabricated by pouring a PDMS mixture (PDMS elastomers and curing agents in a 10:1 weight ratio) into a Petri dish and curing it at 60 °C for 5 h. Two inlets and one outlet were punched using a biopsy punch. The fabricated PDMS layer evenly distributes pressure across the microfluidic chip and fixes the tubing.

The multi-spiral microfluidic chip 4 (MSMC4), which contains four parallel spiral microfluidic channels, consists of three PFPE-PEGDA sheet layers, along with a PDMS top layer for tube fitting. The first layer features an outlet channel for the generated LNPs, while the second layer includes a wide inlet channel for fluid entry and parallel-engraved spiral microfluidic channels where LNPs are formed. The third layer contains two inlets for fluid injection and one outlet for the generated LNPs. Each pattern was engraved using CO_2_ laser engraving, the same method used for the single-spiral microfluidic chip (SMC), and the top PDMS layer was fabricated using the same method. The MSMC8 consists of four PFPE-PEGDA sheet layers. The first layer contains an outlet channel for the generated LNPs, while the second and third layers feature a wide inlet channel for fluid entry and four parallel-arranged spiral microfluidic channels where LNPs are formed. The fourth layer includes two inlets and one outlet. The fabricated layers including a PDMS layer were aligned and fixed in a metal holder, and then each microfluidic chip was connected at the inlet and outlet with PTFE tubing to the PDMS top layer (1/32 in. I.D. × 1/16 in. O.D.). The microfluidic channel was observed using optical microscopy (IX71, OLYMPUS Co., Ltd., Tokyo, Japan).

### 2.3. Fabrication of LNP with Microfluidic Chips

Ethanol containing 0.2 wt.% DPPC and 0.1 wt.% cholesterol was used as the discontinuous phase, while PBS was used as the continuous phase. The flow rate ratio of the discontinuous and continuous phases was fixed at 1:3, and the total flow rate was varied at 30, 60, 90, and 120 mL/h in SMC. For MSMC4, LNPs were synthesized at flow rates of 120, 240, 360, and 480 mL/h, while for MSMC8, synthesis was performed at 240, 480, 720, and 960 mL/h. The flow rate per individual spiral pattern was set to be identical to that of SMC. The size distribution and polydispersity index (PDI) of the synthesized lipid nanoparticles (LNPs) were measured using the dynamic light scattering (DLS) method (Zetasizer Nano ZS, Malvern Instruments, Nottingham, UK).

## 3. Results and Discussion

### 3.1. Fabrication and Characterization of the Spiral Microfluidic Chip

**Figure 1:**
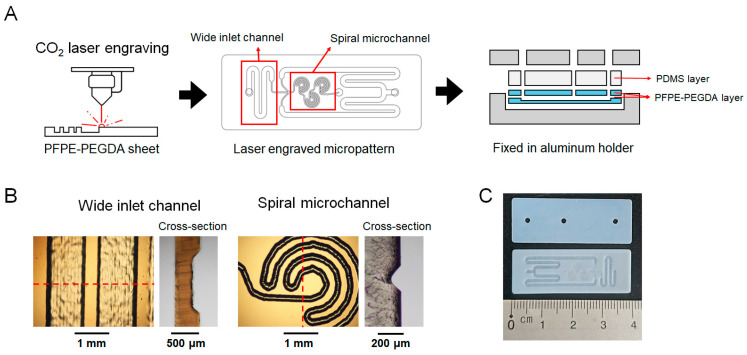
Schematic illustration of (**A**) SMC fabrication procedure and (**B**) OM images of wide inlet channel and spiral microchannel in the first layer of SMC. The red line indicates the cross-sectional area. (**C**) Image of SMC fabricated via laser engraving.

Figure 1A shows a schematic illustration of the spiral microfluidic chip (SMC) fabrication process using the laser engraving method. The first PFPE-PEGDA layer includes a laser-engraved spiral microchannel for rapid mixing and a wide inlet channel for smooth fluid injection. The second layer contains two 1.5 mm diameter inlet holes and one outlet hole, fabricated using laser engraving. The top PDMS layer distributes the pressure applied to the microfluidic chip and is used for PTFE tubing. Figure 1B shows the top view and cross-sectional optical microscopy images of the wide inlet channel and spiral microchannel in the first layer. The width of the wide inlet channel was adjusted to 1354.75 ± 49.31 µm, with a depth of 198.25 ± 15.20 µm. The spiral microchannel was designed to be narrower, with a width of 241.25 ± 17.17 µm and a depth of 147.50 ± 20.86 µm. The wide inlet channel reduces resistance to fluid injection, ensuring smooth entry, and the narrow channel increases the surface area of the two fluids and the flow velocity, enhancing efficient fluid mixing [30,31].

**Figure 2:**
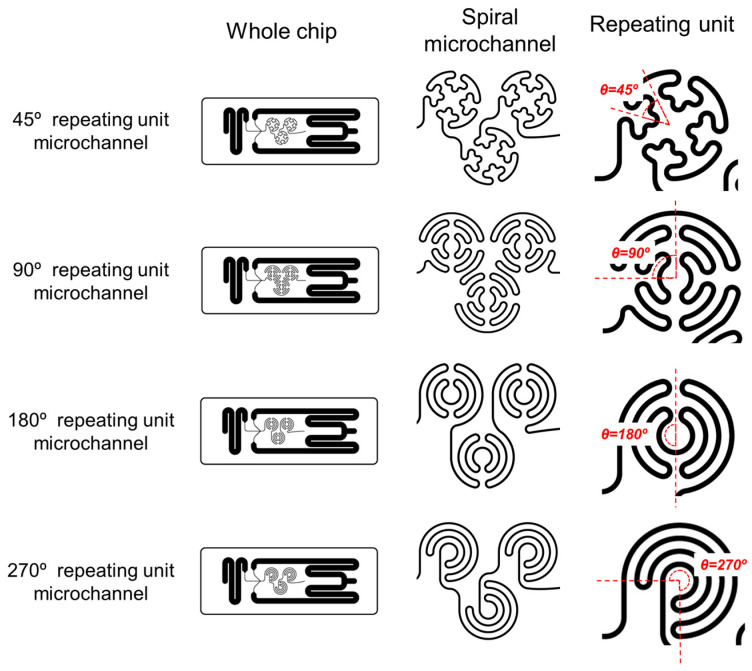
Schematic illustration of spiral microfluidic chip (SMC) designs with varying spiral configurations. The left column shows the overall chip layout, the middle column shows the detailed spiral microchannel structures, and the right column highlights the corresponding repeating unit’s angles (θ = 45°, 90°, 180°, and 270°). These spiral structures influence fluid mixing and LNP formation by modulating the curvature and flow dynamics within the channels.

Figure 2 shows a schematic illustration of various types of SMCs. All SMCs were designed with dimensions of 15 × 40 mm. The spiral micropattern in the first layer of the SMC consists of a spiral channel repeated three times, and four different types of SMCs were fabricated based on the spiral structure. Each spiral structure consists of circular channels with repeating units of 45°, 90°, 180°, and 270°. These curved structures induce centrifugal forces and pressure differences between the inner and outer regions of the channel, generating Dean flow, which facilitates uniform mixing [32,33]. In addition, the laser engraving method enables rapid chip fabrication and easy design modifications, making it suitable for manufacturing and testing various chip designs.

### 3.2. Production and Characterization of Lipid Nanoparticles Using SMC

**Figure 3:**
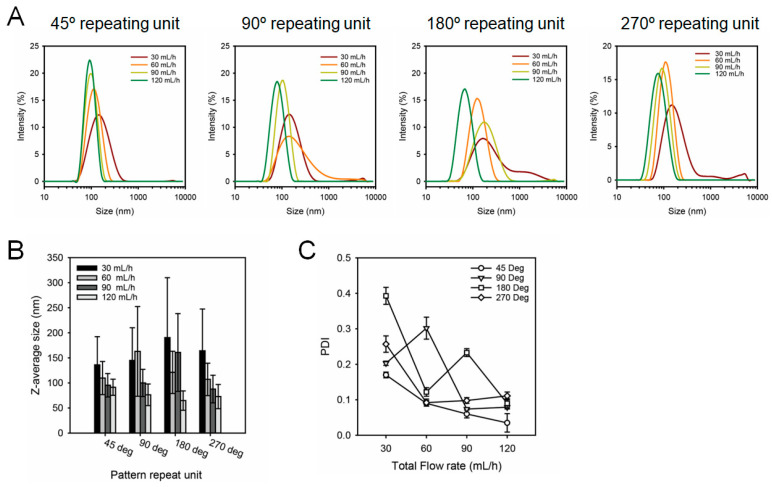
Characterization of lipid nanoparticles (LNPs) produced using spiral microfluidic chips (SMCs) with varying repeating units in a spiral microchannel of SMC (θ = 45°, 90°, 180°, and 270°) at different total flow rates (30, 60, 90, and 120 mL/h). (**A**) Size distribution of LNPs analyzed by dynamic light scattering for each SMC. (**B**) Z-average size graph of LNPs by repeating units and TFR. (**C**) Polydispersity Index (PDI) of LNPs at different flow rates for each SMC.

To evaluate the LNP fabricated using SMCs, lipid nanoparticles were synthesized using DPPC, cholesterol, and PBS. The flow rate ratio (FRR) of the discontinuous phase, containing DPPC and cholesterol, and the continuous phase (PBS) was fixed at 1:3. The experiments were introduced at total flow rates (TFR) of 30, 60, 90, and 120 mL/h. Figure 3A–C show the size distribution, Z-average size, and PDI of LNPs produced using the four types of SMCs. Independent of the repeating unit, all types of SMC demonstrated a reduction in the Z-average size and PDI as the flow rate increased. Figure 3A shows that the size distribution peak shifts to the left with an increase in flow rate across all SMCs, indicating that particle size can be controlled by adjusting the flow rate. Figure 3B,C present the Z-average and PDI of LNPs produced at various flow rates, respectively. At a TFR 120 mL/h, the Z-average sizes produced using SMCs with repeating units of 45°, 90°, 180°, and 270° were 91.35 ± 16.14 nm, 76.34 ± 21.47 nm, 64.72 ± 19.34 nm, and 72.82 ± 24.14 nm, respectively. Independent of the repeating unit, all SMCs generated LNPs with an average particle size below 100 nm at 120 mL/h, and the corresponding PDI values were 0.035 ± 0.026, 0.079 ± 0.005, 0.090 ± 0.013, and 0.111 ± 0.011, demonstrating the production of highly uniform LNPs. At a flow rate of 120 mL/h, the Reynolds number inside the spiral microchannel was calculated using the following equation:

$$Re=\frac{uD_{h}}{\upsilon }$$

where *u* is the average velocity, *D_h_* is the hydraulic diameter, and *υ* is the kinematic viscosity of the fluid. The Reynolds number at the highest flow rate is 214.60, indicating a laminar flow, suggesting that the reduction in LNP particle size and PDI is not caused by vortex-induced mixing but rather by the unique structural characteristics of the SMC.

### 3.3. Fabrication of MSMC for the Mass Production of LNPs

**Figure 4:**
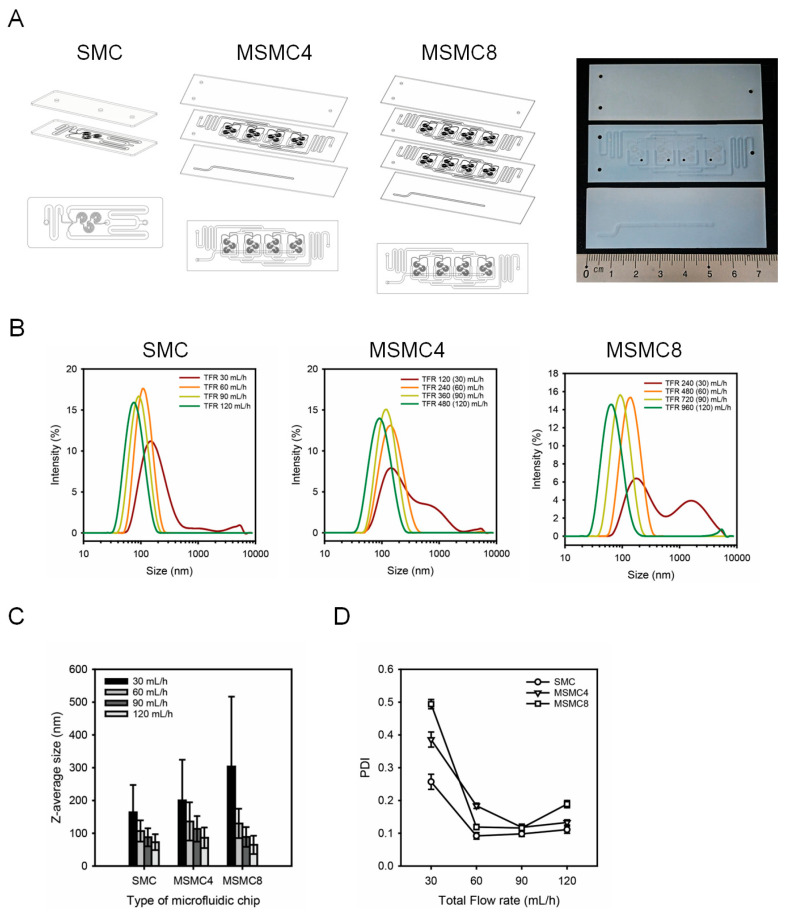
Design and characterization of single- and multi-spiral microfluidic chips (SMC, MSMC4, and MSMC8) for lipid nanoparticle (LNP) production. The repeating unit of the spiral micropattern was fixed at 270°. (**A**) Schematic illustration of the structure of SMC, MSMC4 (four parallel spiral micropatterns), and MSMC8 (eight parallel spiral micropatterns), and an image of MSMC4 fabricated by laser engraving. (**B**) Size distribution of LNPs produced at different total flow rates (TFR) for each microfluidic chip type, measured by dynamic light scattering (DLS). The numbers in brackets represent the average flow rate per individual spiral micropattern. (**C**) Z-average size of LNPs for different chips at various flow rates. (**D**) PDI graph of LNPs by TFR, demonstrating the impact of chip design on the uniformity and scalability of nanoparticles.

In this study, a multi-spiral microfluidic chip (MSMC) was developed to overcome the low productivity of conventional microfluidic devices by arranging spiral micropatterns in parallel. The MSMC consists of at least three PFPE-PEGDA layers and, unlike the SMC, includes a bottom layer with an outlet channel. Based on the Z-average size and PDI produced using the SMC (Table 1), the 270° repeating unit, which allowed for size and PDI control across different flow rates, was adopted for the multi-spiral microchannel design. Microfluidic chips with four parallel spiral micropatterns were labeled as MSMC4, while those with eight channels were labeled as MSMC8. The MSMC8 consists of four PFPE-PEGDA layers, with an additional layer that is structurally identical to the second layer of MSMC4 placed above the outlet layer, facilitating ease of fabrication and upscaling. The total size of the MSMC4 and MSMC8 is 25 × 75 mm (Figure 4A). Figure 4B presents the particle size distributions of LNPs produced using SMC, MSMC4, and MSMC8. The TFR at MSMC4 varied between 120, 240, 360, and 480 mL/h, while for MSMC8, it varied between 240, 480, 720, and 960 mL/h. These flow rates were adjusted to ensure that each spiral micropattern received the same flow rate as in the SMC. Figure 4C shows the Z-average sizes of LNPs produced using SMC, MSMC4, and MSMC8. The LNPs produced using SMC showed Z-average sizes of 164.2 ± 83.22 nm, 107.1 ± 32.37 nm, 87.82 ± 27.52 nm, and 72.82 ± 24.14 nm across increasing flow rates. The LNPs produced using MSMC4 had Z-average sizes of 200.2 ± 124.3 nm, 136.2 ± 58.28 nm, 113.5 ± 38.98 nm, and 86.22 ± 31.43 nm, while those produced using MSMC8 had sizes of 303.5 ± 213.3 nm, 130.1 ± 44.85 nm, 88.77 ± 30.16 nm, and 64.55 ± 28.05 nm, respectively (Table 2). For all three chips, an increase in flow rate resulted in a decrease in LNP particle size, indicating that the parallel arrangement of spiral micropatterns did not affect LNP quality. Although the PDI of LNPs produced using MSMC8 at 120 mL/h showed a slight increase, it remained below 0.2, indicating the production of highly uniform nanoparticles.

## 4. Conclusions

In this study, we developed a microfluidic chip to produce small and uniformly sized LNPs using PFPE-PEGDA polymer and a laser engraving method. Laser engraving offers a cost-effective alternative to conventional microfluidic chip fabrication methods, enabling the rapid fabrication of a single chip in less than five minutes. Although the fabrication of microfluidic chips using laser engraving is limited when creating complex 3D structures, multilayer assembly allowed for MSMCs with parallel configurations to be constructed, thereby enhancing the scalability of the microfluidic system. The base material, PFPE-PEGDA, was fabricated via UV curing, has high plasticity, and is easy to process. The addition of PEGDA enhances hydrophilicity and provides anti-fouling properties.

The spiral micropattern, specifically designed for LNP production, incorporates a unique helical arrangement that enables precise LNP size control and ensures a uniform particle distribution. In MSMC8, which integrates eight parallel spiral micropatterns, it supports a maximum flow rate of 960 mL/h (16 mL/min), enabling high-throughput LNP production while preserving particle quality. The MSMC-based LNP fabrication technology provides high reproducibility, rapid and cost-effective fabrication, and enhanced accessibility for mRNA vaccine and LNP production. This approach presents a viable alternative to the conventional microfluidic chips used for LNP production, offering enhanced efficiency and scalability.

## Figures and Tables

**Table 1:** Z-average size and PDI data of LNPs at different flow rates for each SMC varying repeating unit (θ = 45°, 90°, 180°, and 270°).

Repeating Unit		TFR (mL/h)	30	60	90	120
	Parameter					
45°	Z-average size (nm)		136.1 ± 56.16	109.7 ± 32.99	95.34 ± 23.35	91.35 ± 16.14
	PDI		0.170 ± 0.008	0.090 ± 0.008	0.060 ± 0.011	0.035 ± 0.026
90°	Z-average size (nm)		144.8 ± 65.22	162.9 ± 89.49	100.0 ± 27.18	76.34 ± 21.47
	PDI		0.203 ± 0.006	0.302 ± 0.031	0.074 ± 0.003	0.079 ± 0.005
180°	Z-average size (nm)		190.5 ± 119.4	121.1 ± 42.17	160.8 ± 77.61	64.72 ± 19.34
	PDI		0.393 ± 0.024	0.122 ± 0.012	0.233 ± 0.011	0.090 ± 0.013
270°	Z-average size (nm)		164.2 ± 83.22	107.1 ± 32.37	87.82 ± 27.52	72.82 ± 24.14
	PDI		0.257 ± 0.023	0.092 ± 0.010	0.098 ± 0.008	0.111 ± 0.011

**Table 2:** Z-average size and PDI data of LNPs at SMC, MSMC4, MSMC8 at different flow rates. The repeating unit was fixed at 270°.

Types of Chips		TFR (mL/h)	30	60	90	120
	Parameter					
SMC	Z-average size (nm)		164.2 ± 83.22	107.1 ± 32.37	87.82 ± 27.52	72.82 ± 24.14
	PDI		0.257 ± 0.023	0.092 ± 0.010	0.098 ± 0.008	0.111 ± 0.011
MSMC4	Z-average size (nm)		200.2 ± 124.3	136.2 ± 58.28	113.5 ± 38.98	86.22 ± 31.43
	PDI		0.386 ± 0.023	0.184 ± 0.008	0.118 ± 0.010	0.133 ± 0.008
MSMC8	Z-average size (nm)		303.5 ± 213.3	130.1 ± 44.85	88.77 ± 30.16	64.55 ± 28.05
	PDI		0.494 ± 0.014	0.119 ± 0.008	0.116 ± 0.008	0.189 ± 0.011

## Data Availability

Data are contained within the article.
